# *TP53* mutation characteristics in therapy-related myelodysplastic syndromes and acute myeloid leukemia is similar to *de novo* diseases

**DOI:** 10.1186/s13045-015-0139-z

**Published:** 2015-05-08

**Authors:** Chi Young Ok, Keyur P Patel, Guillermo Garcia-Manero, Mark J Routbort, Jie Peng, Guilin Tang, Maitrayee Goswami, Ken H Young, Rajesh Singh, L Jeffrey Medeiros, Hagop M Kantarjian, Rajyalakshmi Luthra, Sa A Wang

**Affiliations:** Department of Hematopathology, The University of Texas MD Anderson Cancer Center, 1515 Holcombe Boulevard, Houston, TX USA; Department of Leukemia, The University of Texas MD Anderson Cancer Center, 1515 Holcombe Boulevard, Houston, TX USA; Department of Hematology, Central South University Xiangya Hospital, Changsha, China

**Keywords:** *TP53*, Therapy-related, MDS, AML, Karyotype

## Abstract

**Background:**

*TP53* mutation is more prevalent in therapy-related myeloid neoplasms (t-MN) than their *de novo* counterparts; however, the pattern of mutations involving *TP53* gene in t-MN versus *de novo* diseases is largely unknown.

**Methods:**

We collected 108 consecutive patients with therapy-related myelodysplastic syndrome (t-MDS)/acute myeloid leukemia (t-AML). Clinical, hematological, and cytogenetic data were collected by searching the electronic medical record. *TP53* sequencing was performed in all patients using a clinically validated next-generation sequencing-based gene panel assay. A previously published patient cohort consisting of 428 patients with *de novo* MDS/AML was included for comparison.

**Results:**

We assessed 108 patients with t-MN, in which 40 patients (37%) had *TP53* mutations. The mutation frequency was similar between t-MDS and t-AML; but significantly higher than *de novo* MDS/AML (62/428 patients, 14.5%) (*p* < 0.0001). *TP53* mutations in t-MN were mainly clustered in DNA-binding domains, with an allelic frequency of 37.0% (range, 7.1 to 98.8). Most mutations involved single nucleotide changes, of which, transitions (65.9%) were more common than transversions (34.1%). Missense mutations were the most frequent, followed by frameshift and nonsense mutations. This *TP53* mutation pattern was strikingly similar to that observed in *de novo* MDS/AML. *TP53* mutations in t-MN were associated with a complex karyotype (*p* < 0.0001), a higher number of chromosomal abnormalities (*p* < 0.0001), and an inferior overall survival in affected patients (6.1 vs 14.1 months) by univariate (*p* < 0.0001) and multivariate analyses (*p* = 0.0020).

**Conclusions:**

Our findings support the recent notion that heterozygous *TP53* mutation may be a function of normal aging and that mutated cells are subject to selection upon exposure to cytotoxic therapy. t-MN carrying *TP53* mutation have an aggressive clinical course independent of other confounding factors.

## Introduction

Therapy-related myeloid neoplasms (t-MN) are a group of hematopoietic myeloid neoplasms occurring in patients who previously received various cytotoxic chemotherapy regimens and/or radiation therapy for cancer, or rarely, non-neoplastic diseases [1]. Under the current WHO classification, t-MN is considered in a single category because these diseases invariably have a dismal outcome although morphologic variants, such as therapy-related myelodysplastic syndrome (t-MDS), therapy-related acute myeloid leukemia (t-AML), and therapy-related myelodysplastic syndrome/myeloproliferative neoplasm (t-MDS/MPN) are recognized [2,3]. The incidence of t-MN after chemotherapy and/or radiotherapy has been estimated to be 0.8% and 6.3% at 20 years [4]. t-MN are a group of clinically aggressive diseases and respond poorly to conventional therapies with rapid disease progression [5,6]. Chromosomal abnormalities are observed in up to 80%–90% t-MN patients with frequent high-risk cytogenetic abnormalities [7,8]. Our group recently showed that t-MN has a mutational profile distinct from *de novo* MDS/AML [9].

p53 protein, encoded by *TP53*, is a tumor suppressor protein that consists of transactivation domain, proline-rich domain, DNA-binding domain, oligomerization domain, and regulatory domain [10]. p53 responds to diverse cellular stresses to induce cell cycle arrest, apoptosis, and DNA repair. Somatic *TP53* mutations are found in a variety of cancers with various frequencies depending on cancer type [11]. Most *TP53* mutations are clustered in the DNA-binding domain encompassing exons 5 and 8, and most mutations (87.9%) in the DNA-binding domain are missense mutations [11]. Overall, *TP53* mutations are found in 5% ~ 10% of *de novo* MDS and AML and were shown to be associated with a complex karyotype and shorter survival [12-14]. In contrast, *TP53* mutations are found in 21%–38% t-MN and are associated with 5q-, a complex karyotype and a poor prognosis [15-17].

Recently, Wong and colleagues sequenced the genomes of 22 patients with t-AML and showed that the total number of somatic single-nucleotide variants and the percentage of chemotherapy-related transversions were similar in t-AML and *de novo* AML [18]. These findings indicate that cytotoxic therapy does not induce genome-wide DNA damage, nor does cytotoxic therapy directly induce *TP53* mutations. In four t-AML patients with *TP53* mutation, the exact mutation in *TP53* genes was found at a low frequency (0.003%–0.7%) in mobilized blood leukocytes or bone marrow 3–6 years before the development of t-AML/t-MDS. Additionally, *TP53* mutations at a low frequency were found in elderly healthy individuals. These findings indicate that *TP53* mutations may be age related and that cells carrying this mutation might be resistant to chemotherapy and expand preferentially after treatment. The early acquisition of *TP53* mutations in the founding stem cell clone probably contributes to the frequent cytogenetic abnormalities and poor responses to chemotherapy that are typical of patients with t-AML/t-MDS.

We conducted this study to compare the mutational characteristics of *TP53* in t-MN and their *de novo* counterparts in a large patient cohort. We also correlated *TP53* mutation status with the results of cytogenetic studies and evaluated the clinical significance of *TP53* mutations in patients with t-MN.

## Results

### Patient characteristics

*TP53* mutation analysis was performed on 108 patients’ bone marrow (BM) whole cell samples, including 53 t-MDS and 55 t-AML. The previous diseases in these patients included: 49 hematological malignancies, 45 carcinomas, 6 sarcomas, 5 germ cell tumors, 1 malignant mixed müllerian tumor, 1 medulloblastoma, and 1 rheumatoid arthritis. Sixty-one patients were treated with chemotherapy, 8 radiation therapy only and 39 with combined chemoradiation therapy. There were 62 men and 46 women with a median age of 68 years (range, 18–87). Karyotypic information was available in 105 patients and an abnormal karyotype was identified in 89 (84.8%) patients, including 56 (53.3%) with a complex karyotype (≥3 abnormalities). The t-MDS cases could be further sub-categorized as refractory cytopenia with unilineage dysplasia (RCUD) (*n* = 1, 1.9%), refractory anemia with ring sideroblasts (RARS) (*n* = 1, 1.9%), refractory cytopenia with multilineage dysplasia (RCMD) (*n* = 24, 45.3%), refractory anemia with excess blasts (RAEB)-1 (*n* = 15, 28.3%), and RAEB-2 (*n* = 12, 22.6%). According to the International Prognostic Scoring System (IPSS) risk categorization, 8 (15.7%), 14 (27.5%), and 29 (56.9%) patients had good, intermediate, and poor cytogenetic risk, respectively. For 55 t-AML, 2 (3.7%), 13 (24.1%), and 39 (72.2%) patients had favorable, intermediate, and adverse cytogenetic risk, respectively, using the revised United Kingdom Medical Research Council (UKMRC) prognostic system.

### *TP53* mutations in t-MDS and t-AML

**Figure 1:**
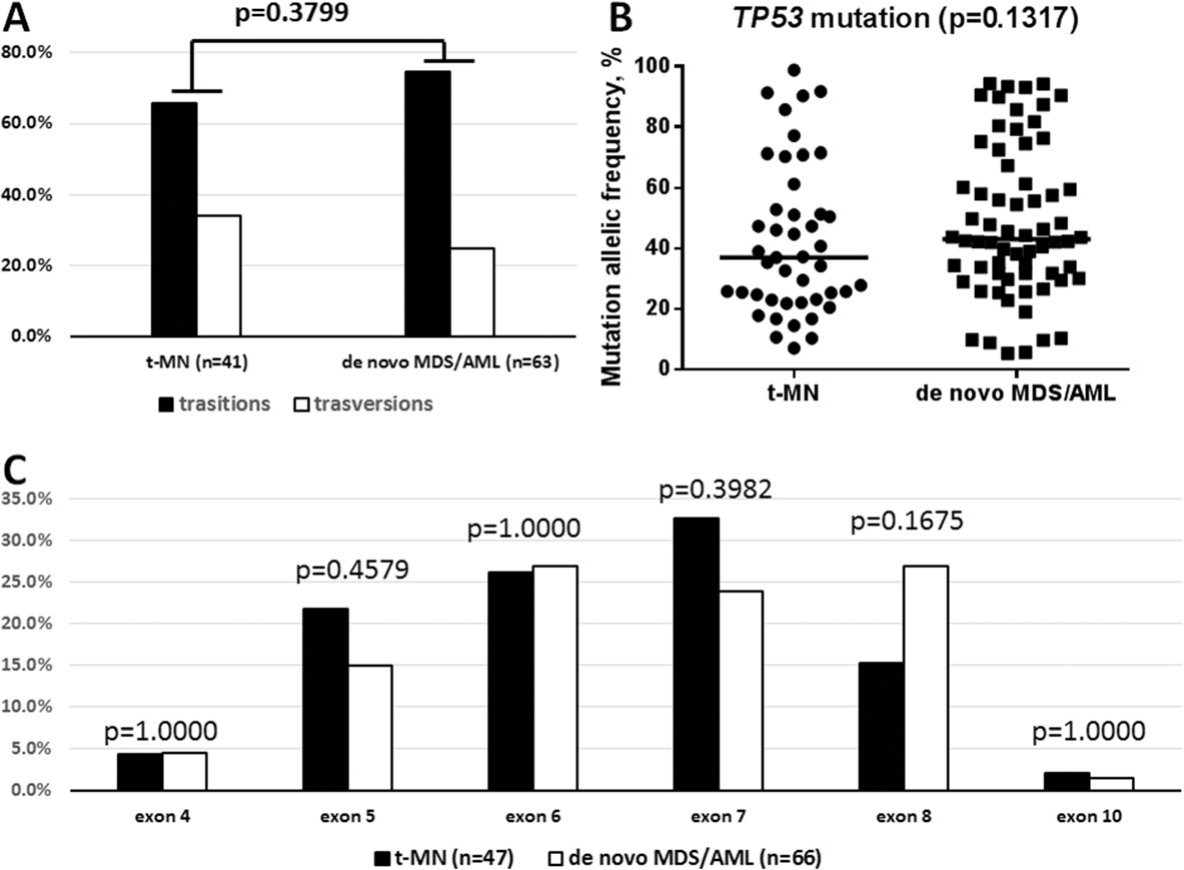
Patterns of *TP53* mutations in t-MN and *de novo* MDS/AML. **(A)** Frequencies of transitions and transversions in t-MN and *de novo* MDS/AML; the numbers in parentheses denote the number of single nucleotide changes in each group. **(B)** *TP53* mutational allelic frequencies in t-MN and *de novo* MDS/AML. **(C)** Loci of *TP53* mutations in t-MN and *de novo* MDS/AML. The numbers in parentheses denote the number of all mutations in each group.

A total of 47 different mutations in *TP53* were detected in 40 of 108 (37%) patients. Thirty-three (*n* = 33) had a single *TP53* mutation, and 7 patients had two *TP53* mutations. There were 41 single nucleotide changes and 6 insertion/deletions (indels). Among single nucleotide changes, there were 27 (65.9%) transitions and 14 (34.1%) transversions (Figure 1A). Classes of single nucleotide changes are described in Table 1. The median mutational allelic burden was 37% (range, 7.1%–98.8%) (Figure 1B). Twenty-nine (29) patients had mutations only involving *TP53* and 11 patients had mutations in other genes including *PTPN11*, *FLT3*, *IDH1*, *NRAS*, *KIT*, *JAK2*, and *MPL*. Most *TP53* mutations were missense (*n* = 38), followed by frameshift (*n* = 5) and nonsense (*n* = 4) mutations. Mutations were widely distributed in exons 4 (*n* = 2), 5 (*n* = 10), 6 (*n* = 12), 7 (*n* = 15), 8 (*n* = 7), and 10 (*n* = 1) (Figure 1C). Codon 248 (*n* = 7) was the most frequently mutated locus.

**Table 1:** **Single nucleotide changes of** ***TP53*** **mutations in therapy-related versus** ***de novo*** **myelodysplastic syndromes/acute myeloid leukemia (MDS/AML)**

**Class**	**t-MN,** ***n*** **(%)**	***De novo*** **MDS/AML,** ***n*** **(%)**
C:G > A:T	5 (12.2)	1 (1.6)
C:G > G:C	3 (7.3)	6 (9.5)
C:G > T:A	18 (43.9)	35 (55.6)
T:A > A:T	4 (9.8)	3 (4.8)
T:A > C:G	9 (22.0)	12 (19.0)
T:A > G:C	2 (4.9)	6 (9.5)
Total	41 (100)	63 (100)

In t-MDS, 26 mutations in *TP53* were identified in 21 of 53 (39.6%) patients. The median mutational allelic burden was 34.9% (range, 10.4–91.7). There were 20 single nucleotide changes, of which transversions (*n* = 11) were more common than transitions (*n* = 9), and indels (*n* = 6). *TP53* mutations in t-MDS were distributed in exons 5 (*n* = 8), 6 (*n* = 8), 7 (*n* = 6), and 8 (*n* = 4). In t-AML, 21 mutations in *TP53* were detected in 19 of 55 (34.5%) patients. The median mutational allelic frequency was 37.3% (range, 7.1–98.8), which was not significantly different from that of t-MDS (*p* = 0.6624). All 21 mutations in t-AML were single nucleotide changes, of which 18 were transitions and 3 were transversions. The proportion of transversions was significantly lower in t-AML compared with t-MDS (11/20 vs 3/21, *p* = 0.0088). *TP53* mutations in t-AML were detected in exons 4 (*n* = 2), 5 (*n* = 2), 6 (*n* = 4), 7 (*n* = 9), 8 (*n* = 3), and 10 (*n* = 1), similar to t-MDS.

### Comparing *TP53* mutations in t-MDS/AML and *de novo* MDS/AML

We compared the current cohort with a previously published cohort [9] of *de novo* MDS/AML (*n* = 428) where *TP53* mutation was detected in 62 (14.5%) patients. In *de novo* MDS/AML, 56 patients had single *TP53* mutation and 6 patients had double *TP53* mutations. Similar to t-MDS/AML, single nucleotide changes (*n* = 63) were the predominant form, followed by four indels and one duplication. Transitions were more frequent (*n* = 47, 74.6%), similar to that in t-MDS/AML (65.9%) (*p* = 0.3799) (Figure 1A). Classes of single nucleotide changes are described in Table 1. The median mutational allelic burden was 43.1% (range, 5.4–94.3), which was not significantly different from that of t-MDS/AML (37.0%, 7.1%–98.8%) (*p* = 0.1317) (Figure 1B). Similar to t-MDS/AML, missense (*n* = 54) was the most common type of mutation, followed by nonsense (*n* = 7), frameshift (*n* = 5), and splice site mutations (*n* = 2). Mutations were detected in exons 4 (*n* = 3), 5 (*n* = 10), 6 (*n* = 18), 7 (*n* = 16), 8 (*n* = 18), and 10 (*n* = 2) (Figure 1C). Comparing mutation distribution in different exons between t-MN and *de novo* AML/MDS, significant difference was not observed in each exon. Fifty-two patients had mutations only in *TP53* and 10 patients had mutations in other genes including *BRAF*, *DNMT3A*, *FLT3*, *IDH1*, *IDH2*, *JAK2*, *KRAS*, and *NPM1*. This mutation frequency was not significantly different from t-MN (10/62 vs 10/39, *p* = 0.3070).

### *TP53* mutation correlated with clinical parameters and cytogenetics

t-MDS/AML with *TP53* mutation showed a significantly lower mean corpuscular volume (MCV) (median, 90 vs. 93, *p* = 0.0076) and platelet counts (median, 32 × 10^9^ vs. 42 × 10^9^/L, *p* = 0.0393) compared with t-MN without *TP53* mutation. There were no significant differences in demographics, hemoglobin level, leukocytes, and absolute neutrophil counts, and prior cytotoxic therapy between patients with mutated or wild-type *TP53*.

**Figure 2:**
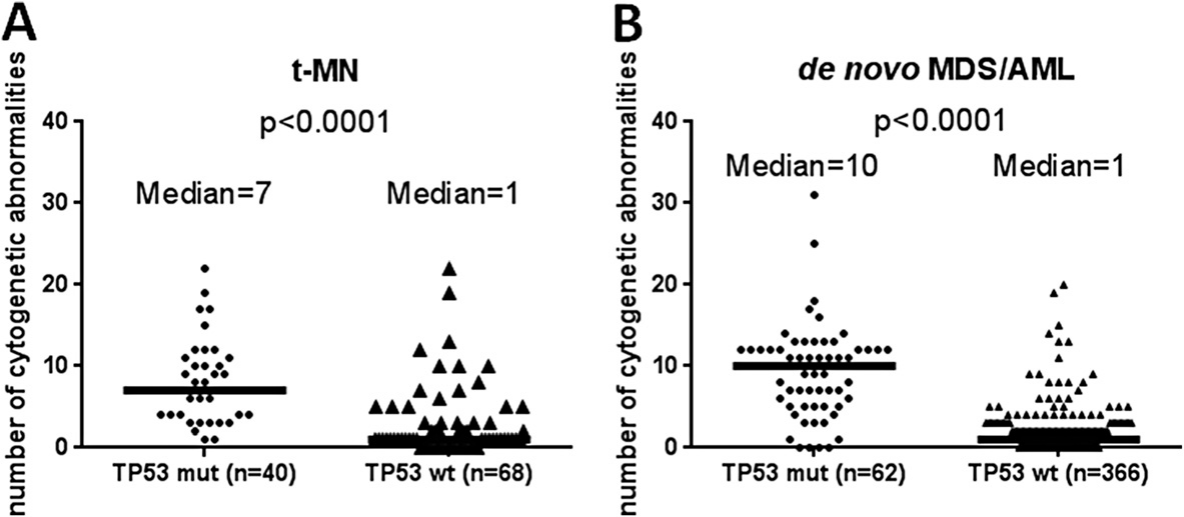
Number of cytogenetic abnormalities with respect to *TP53* mutation in therapy-related myeloid neoplasm **(A)** and in *de novo* myelodysplastic syndromes/acute myeloid leukemia (MDS/AML) **(B)**. TP53 mut, cases with *TP53* mutation; TP53 wt, cases with wild-type *TP53*.

The distribution of cytogenetic data was significantly different between t-MN with and without *TP53* mutation. Overall, a higher cytogenetic risk was more common in t-MN with *TP53* mutation (*p* < 0.0001), particularly, a complex karyotype (*p* < 0.0001) (Table 2). In t-MDS/AML with a diploid or a non-complex aberrant karyotype (*n* = 49), *TP53* mutation was detected in only three (6.1%) patients. *TP53* mutations were associated with frequent -5/-5q abnormalities (71.8% vs. 26.9%, *p* < 0.0001) but not -7/-7q abnormalities (*p* = 0.2067). When all numerical and structural chromosomal abnormalities were counted, *TP53* mutation was shown not only to correlate with a complex karyotype but also the total number of karyotypic abnormalities (*P* < 0.0001) (Figure 2A, B), in both t-MN and *de novo* MDS/AML.

**Table 2:** **Comparison between therapy-related myeloid neoplasm with and without** ***TP53*** **mutation**

	**Mutated** ***TP53*** **(** ***n*** **= 40)**	**Wild-type** ***TP53*** **(** ***n*** **= 68)**	***p*** **value**
Age, years, median (range)	66 (23–87)	68 (18–82)	0.6491*
Male: female	22:18	40:28	0.8405
Prior therapy			
Chemotherapy only	21	40	0.5518
Radiation only	2	6	0.7082
Combined chemoradiation	17	22	0.2972
Hemoglobin, g/L, median (range)	91 (66–1128)	98 (60–139)	0.1775*
Mean corpuscular volume, median (range)	90 (70–107)	93 (79–116)	0.0076*
White blood cell, × 10^9^/L, median (range)	3.0 (0.3–41)	3.0 (0.6–93.9)	0.5314*
Absolute neutrophil count, × 10^9^/L, median (range)	0.8 (0–9.2)	1.1 (0–30.62)	0.2223*
Platelet, × 10^9^/L, median (range)	32 (5–394)	42 (7–364)	0.0393*
Cytogenetic data			<0.0001
Diploid	0	16	
Non-complex (<3)	3	30	
Complex (≥3)	36	20	
Not available	1	2	
Chromosome 5 aberrations	28	14	<0.0001
Chromosome 7 aberrations	18	23	0.2067
Number of abnormalities, median (range)	7 (1–22)	1 (0–22)	<0.0001*

Asterisk (*) denotes *p* value was calculated by Mann-Whitney *U* test. All the others were calculated by Fisher’s exact test.

### Prognostic significance of *TP53* mutations in t-MN

**Figure 3:**
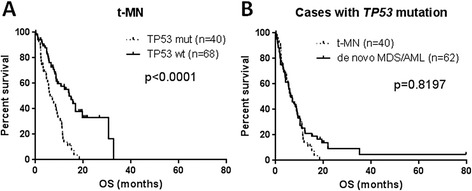
OS comparison. Overall survival (OS) comparison with respect to *TP53* mutation in patients with therapy-related myeloid neoplasm (t-MN) **(A)**. OS comparison between t-MN with *TP53* mutation and *de novo* MDS/AML with *TP53* mutation **(B)**. TP53 mut, patients with *TP53* mutation; TP53 wt, patients with wild-type *TP53*.

We collected survival data with a median follow-up of 12.9 months including alive and dead patients (range, 0.1–198.3). After censoring patients who received stem cell transplant (SCT) at the time of the procedure, the median overall survival (OS) of all t-MDS/AML patients was 8.0 months (range, 0.1–32.7). t-MDS/AML patients with *TP53* mutation showed a significantly shorter median OS (6.1 months) compared with patients with wild-type *TP53* (14.1 months) (*p* < 0.0001) (Figure 3A). Of patients with *TP53* mutations, the OS was very similar between patients with t-MN or *de novo* MDS/AML (6.2 months, range 0.2–79.7) (*p* = 0.8197) (Figure 3B). The location (exons) or type (transition/transversion) of *TP53* mutations did not correlate with OS (*p* = 0.2922 and *p* = 0.9209, respectively). In univariate analysis, hemoglobin level (<10 g/dL), and platelet count (<50 × 10^9^/L), a complex karyotype and *TP53* mutation were identified as significant hazards. In multivariate analysis, platelet count, male gender and *TP53* mutation (*p* = 0.002) remained to be independent hazards (Table 3).

**Table 3:** **Overall survival of patients with therapy-related myeloid neoplasm by univariate and multivariate analysis**

**Variables**	**Univariate analysis**			**Multivariate analysis**		
	**HR**	**95% CI**	***p*** **value**	**HR**	**95% CI**	***p*** **value**
Age (>60 years)	1.001	0.572–1.753	0.997	0.995	0.553–1.792	0.988
Gender (man)	0.708	0.438–1.143	0.157	0.541	0.315–0.927	*0.025*
ANC (<0.8 × 10^9^/L)	1.313	0.821–2.099	0.256	0.820	0.482–1.392	0.462
BM blast (≥5%)	1.633	0.941–2.836	0.081	1.580	0.853–2.924	0.145
Hemoglobin (<100 g/L)	1.946	1.153–3.286	*0.013*	1.711	0.950–3.081	0.073
Platelet (<50 × 10^9^/L)	2.181	1.291–3.683	*0.004*	2.023	1.153–3.549	*0.014*
Complex karyotype	2.878	1.694–4.888	*<0.001*	1.150	0.546–2.425	0.713
*TP53* mutation	2.971	1.823–4.841	*<0.001*	2.958	1.476–5.929	*0.002*

Abbreviations: HR, hazard ratio; CI, confidence interval; ANC, absolute neutrophil count; BM, bone marrow.

*p* value < 0.05 is demonstrated in *italics*.

## Discussion

In this study, we show that t-MN carry a high frequency of *TP53* mutation than their *de novo* counterparts. However, the mutation allele frequency, nucleotide alterations, and mutation patterns in *TP53* are not different between MDS/AML patients with or without a history of cytotoxic exposure. *TP53* mutation in t-MN is an independent risk for an inferior outcome.

In this cohort of 108 t-MN patients, *TP53* mutation was detected in 37% of patients. This frequency of *TP53* mutations was comparable to a frequency of 20% ~ 40% reported by others [15,17,19]. Most mutations were missense mutations and clustered in the DNA-binding domain (exons 5–8). The most common mutation type was C:G to T:A substitution (43.9%), and this frequency was similar to the data reported by Wong and colleagues of approximately 50% [18]. The most frequent mutated locus in t-MN was codon 248, which is one of the most frequently mutated codons (reported in COSMIC (http://cancer.sanger.ac.uk)) as well as in IARC *TP53* database (R17, November 2013). Mutations in this codon of *TP53* have been reported in colorectal cancer, laryngeal squamous cell carcinoma, prostate cancer, breast cancer, and ovarian cancer [20].

We show that the pattern of *TP53* mutation and mutational allelic frequency in t-MN are similar to that in *de novo* MDS/AML. The lack of unique features of mutation in *TP53* gene between t-MN and *de novo* diseases indicates that cytotoxic therapy does not cause genome-wide damage in *TP53*; rather the genetic insult to *TP53* gene is similar in MDS/AML either secondary to cytotoxic therapy or occurring *de novo*. The former concept was supported by the study of Wong and colleagues [18] who showed that *TP53* mutations detected at the diagnosis of t-AML were also found in mobilized blood leukocytes or bone marrow 3–6 years prior to the diagnosis of t-MN in the same patients. They proposed a model of t-AML harboring clonal *TP53* mutation that somatic *TP53* mutations are present in the hematopoietic stem cells (HPSC) of some healthy individuals; and these HPSC may expand under the selective pressure of chemotherapy. The findings derived from this large patient cohort in this study endorse this disease model.

Our group has shown that using NGS methods, the mutational profile t-MDS and t-AML, are different [9]. The overall mutation frequency and the number of involved genes were significantly higher in t-AML than t-MDS. However, with respect to *TP53*, mutations were equally frequent in t-AML and t-MDS. In this study, with this expanded patient cohort, not only did we further confirm our previous observation but also showed that the allele frequency and mutation pattern of *TP53* were nearly identical in t-MDS and t-AML. Interestingly, 5 of 11 (45.5%) transversions detected in t-MDS were C:G > A:T substitution. This specific substitution was detected in 1 of 7 (14.3%) transversions in *de novo* MDS and was not detected in t-AML or *de novo* AML. The prevalence of such specific mutations was reported to be significantly higher in smoking-associated lung cancers compared to lung cancers of non-smokers [21]. However, it was also reported that the frequencies of transitions versus transversions as well as the specific type of transitions/transversion of *TP53* mutations in breast cancer patients differed by ethnic background and had no clinical significance [22]. It is uncertain at this point if this specific transversion was more prevalent in MDS over AML. Nevertheless, our findings in t-MDS vs t-AML provide further support to the model that *TP53* mutation is an early event in pathogenesis of t-MN, and additional molecular genetic events, particularly mutations in class I genes, likely provide proliferative advantage in cases of t-AML.

Similar to what has been reported previously [17,19,23], *TP53* mutations were highly associated with a complex karyotype and frequent deletions involving chromosome 5 in t-MN. In this study, we further showed that *TP53* mutation correlated with a higher number of structural and numerical chromosomal abnormalities in t-MN. On the other hand, *TP53* mutations were identified in approximately 5% of cases with a normal or a non-complex karyotype in t-MN. It is likely that p53 dysfunction leads to genome instability and facilitates cytogenetic complexity. It is noteworthy that approximately 40% of t-MN cases with a complex karyotype had no *TP53* mutations, suggesting that other factors, probably multiple cytotoxic insults, may contribute to karyotypic complexity. We also showed that *TP53* mutation in t-MN predicts a shorter overall survival in t-MN, and the risk of *TP53* mutation is an independent adverse risk factor. In contrast, a complex karyotype failed to show its independent prognostic value when it was co-analyzed with other confounding factors including *TP53* mutation status.

## Conclusions

In summary, t-MDS and t-AML both harbor a high frequency of *TP53* mutations, significantly higher than their *de novo* counterpart. However, the mutation type, pattern, distribution of mutated loci, and mutational allelic frequency in *TP53* are neither different between therapy-related and *de novo* MDS/AML nor between t-MDS and t-AML. These findings support the recent model proposed by Wong and colleagues that *TP53* mutation occurs at a very early stage of leukemogenesis of t-MN, but other factors likely contribute to further development of clinically and histopathologically evident t-MN. Overall, *TP53* mutation in t-MN is strongly associated with a complex karyotype as well as the number of karyotypic abnormalities. *TP53* mutation predicts a poorer survival and is an independent adverse risk factor in patients with t-MN.

## Methods

### Patients

We collected 108 consecutive patients with therapy-related MDS/AML from October 2012 through January 2014 at the University of Texas MD Anderson Cancer Center. Clinical, hematological, and cytogenetic data were collected by searching the electronic medical record. The types of primary malignant or non-malignant diseases for which cytotoxic therapy was administered were also collected. Brachytherapy, radioisotopes, and radiation therapy in patients in whom the field did not include active hematopoietic bone marrow were not considered as radiation therapy. All cases were collected consecutively and classified according to the World Health Organization (WHO) classification system. A previously published patient cohort consisting of 428 patients with *de novo* MDS/AML was included for comparison [9]. This study was conducted in accord with the Declaration of Helsinki and was approved by the IRB at the University of Texas MD Anderson Cancer Center in Houston, TX, USA.

### *TP53* sequencing

*TP53* sequencing was performed in all patients using a clinically validated 53-gene panel or a 28-gene panel assay. The 53-gene panel covers exons (codons) 2, 4–8, and 10 (1–12, 69–112, 126–253, 267–206, and 332–342) and the 28-gene panel covers exons (codons) 4–10 (41–224 and 234–367). Briefly, genomic DNA (gDNA) was extracted from bone marrow aspirate or peripheral blood of each case using an Autopure extractor (Qiagen, Valencia, CA, USA). A sequencing library was prepared using 250 ng of DNA template and either 53- or 28-gene panel. The sequencing library was purified using AMPure magnetic beads (Agencourt, Brea, CA, USA) and then subjected to MiSeq sequencer (Illumina Inc., San Diego, CA, USA) [24]. A minimum quality score of AQ30 is required for a minimum of 75% of bases sequenced ensuring high-quality sequencing results. Variant calling was performed with Illumina MiSeq Reporter Software 1.3.17 using human genome build 19 (hg 19) as a reference and sequencing reads were aligned using the Integrative Genomics Viewer (IGV, Broad Institute, MA, USA) [25]. For clinical reporting, a sequencing coverage of 250× (bi-directional true paired-end sequencing) and a variant frequency of 5% in a background of wild-type *TP53* were used as cutoffs.

### Cytogenetic analysis

Conventional cytogenetic analysis was performed using standard methods as described previously [26]. Twenty metaphases were analyzed, and the results were reported using the current International System for Human Cytogenetic Nomenclature [27]. Only karyotypes with adequate metaphases for analysis were included, except in some cases where lesser numbers of metaphases were available, fluorescence *in situ* hybridization (FISH) was performed to confirm clonal cytogenetic abnormalities. For MDS patients, the cytogenetic risk was stratified according to the IPSS [28]; and for AML patients, the risk was categorized by the revised cytogenetic classification proposed by the UKMRC [29].

### Statistical analysis

For continuous variables, data were reported as a median and range. For nominal variables, data were reported as the number of patients if not otherwise specified. Fisher’s exact test and the Mann-Whitney *U* test were used for categorical variables and for continuous variables, respectively. OS was calculated from the day of diagnosis to the last follow-up. For patients who received hematopoietic stem cell transplant (HSCT), survival was censored at the day of the procedure. Distributions of OS were estimated by Kaplan and Meier curves and survival differences were evaluated using the log-rank test. All differences with *p* < 0.05 were considered to be statistically significant (two-tailed). GraphPad Prism 6.0 (La Jolla, CA, USA) and SPSS V22 (Armonk, NY, USA) were used for statistical analyses.
